# Development of mandibular, hyoid and hypobranchial muscles in the zebrafish: homologies and evolution of these muscles within bony fishes and tetrapods

**DOI:** 10.1186/1471-213X-8-24

**Published:** 2008-02-28

**Authors:** Rui Diogo, Yaniv Hinits, Simon M Hughes

**Affiliations:** 1 MRC Centre for Developmental Neurobiology and Randall Division for Cell and Molecular Biophysics, New Hunt's House, King's College London SE1 1UL, UK; 2 Department of Anthropology, George Washington University, USA

## Abstract

**Background:**

During vertebrate head evolution, muscle changes accompanied radical modification of the skeleton. Recent studies have suggested that muscles and their innervation evolve less rapidly than cartilage. The freshwater teleostean zebrafish (*Danio rerio*) is the most studied actinopterygian model organism, and is sometimes taken to represent osteichthyans as a whole, which include bony fishes and tetrapods. Most work concerning zebrafish cranial muscles has focused on larval stages. We set out to describe the later development of zebrafish head muscles and compare muscle homologies across the Osteichthyes.

**Results:**

We describe one new muscle and show that the number of mandibular, hyoid and hypobranchial muscles found in four day-old zebrafish larvae is similar to that found in the adult. However, the overall configuration and/or the number of divisions of these muscles change during development. For example, the undivided adductor mandibulae of early larvae gives rise to the adductor mandibulae sections A0, A1-OST, A2 and Aω, and the protractor hyoideus becomes divided into dorsal and ventral portions in adults. There is not always a correspondence between the ontogeny of these muscles in the zebrafish and their evolution within the Osteichthyes. All of the 13 mandibular, hyoid and hypobranchial muscles present in the adult zebrafish are found in at least some other living teleosts, and all except the protractor hyoideus are found in at least some extant non-teleost actinopterygians. Of these muscles, about a quarter (intermandibularis anterior, adductor mandibulae, sternohyoideus) are found in at least some living tetrapods, and a further quarter (levator arcus palatini, adductor arcus palatini, adductor operculi) in at least some extant sarcopterygian fish.

**Conclusion:**

Although the zebrafish occupies a rather derived phylogenetic position within actinopterygians and even within teleosts, with respect to the mandibular, hyoid and hypobranchial muscles it seems justified to consider it an appropriate representative of these two groups. Among these muscles, the three with clear homologues in tetrapods and the further three identified in sarcopterygian fish are particularly appropriate for comparisons of results between the actinopterygian zebrafish and the sarcopterygians.

## Background

**Figure 1:**
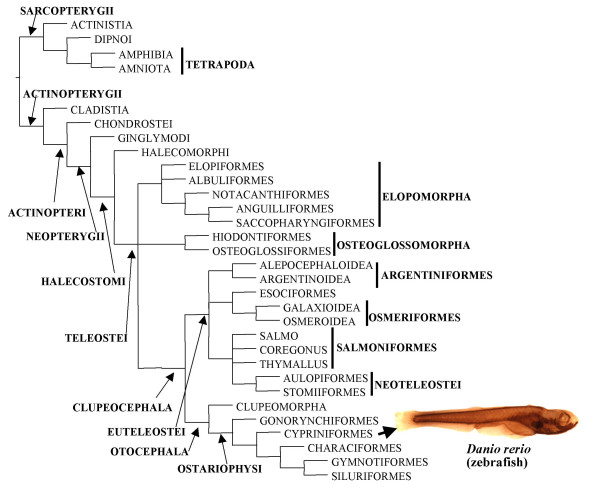
Phylogenetic relationships among the major extant osteichthyan groups (modified from Diogo [36]: the Elopomorpha, Osteoglossomorpha and Clupeocephala are placed in a trichotomy; see also [78]).

The Osteichthyes, including bony fishes and tetrapods, is a highly speciose group of gnathostomes, comprising more than 42,000 living species. Two main osteichthyan groups are usually recognized: the Actinopterygii (rayfins, > 28,000 extant species) and Sarcopterygii (lobefins and tetrapods, > 24,000 living species; note that bony fishes constitute a paraphyletic group, as they only become monophyletic if tetrapods are excluded; Fig. 1) [1]. One of the most studied osteichthyan model organisms is the zebrafish *Danio rerio*, a small actinopterygian freshwater fish from the teleostean order Cypriniformes (Fig. 1) [2-5]. Comparisons between zebrafish and other vertebrates are often made in developmental studies, the zebrafish being sometimes taken as a 'good representative' of teleosts, of actinopterygians and even of bony fishes [2]. To what extent is this true for the cranial musculature?

Among studies dealing with zebrafish myology, only a few focus on cranial muscles, and these mainly concern larval stages (e.g. [3,5-10]). In fact, as stated by Schilling, "no study has carefully described the anatomy of the musculature of the adult zebrafish" [5]. This is surprising given that the cranial myology of other adult members of the order Cypriniformes has been described in detail in the literature [11-20]. Schilling provided a short summary of the myology of the adult zebrafish but, as he recognized, this was mainly based on an extrapolation from his "own observations of larval cranial muscles" and from "studies in other teleosts", and not from direct dissection of adult specimens of *Danio rerio *[5].

Aside from the poor knowledge of the late stages of development of zebrafish cranial muscles there are also problems with the identification of homologies between some of these muscles and those of other vertebrates. The main reason for the scarce knowledge of the homologies and evolution of osteichthyan cranial muscles is that most of the works dealing with the comparative anatomy of Osteichthyes concern skeletal structures. The most detailed published comparative analyses of osteichthyan cranial muscles based on direct observation of a wide range of taxa derive from long ago [12,21-23]. Edgeworth's volume [12] continues to be a fundamental source of information on vertebrate muscles. However, Edgeworth could not, for instance, study the muscles of the then undiscovered coelacanth *Latimeria chalumnae *[24], or know of the essential role of neural crest cells in the development and patterning of vertebrate cranial muscles [3,25-33].

About 15 years ago, Miyake et al. [34] published an analysis of the cranial muscles of chondrichthyan batoids, in which they re-examined and discussed various hypotheses proposed by Edgeworth [12]. They noted that "Noden [26-28] elegantly demonstrated with quail-chick chimeras that (certain) cranial muscles are embryologically of somitic origin and not, as commonly thought, of lateral plate origin, and in doing so corroborated the nearly forgotten work of Edgeworth". They also pointed out that molecular developmental studies such as Hatta et al. [7,35] "have corroborated one of Edgeworth's findings: the existence of one premyogenic condensation (the constrictor dorsalis) in the cranial region of teleost fish". Edgeworth [12] recognized various presumptive premyogenic condensations: mandibular, hyoid, branchial, epibranchial, and hypobranchial. According to him, developmental pathways leading from these condensations to myogenesis in each cranial arch involve migration of premyogenic cells, differentiation of myofibers, directional growth of myofibers and possibly interactions with surrounding structures. These events occur in very specific locations, e.g. dorsal, medial or ventral areas of each arch. Although exceptions may occur [36], Edgeworth's mandibular muscles are generally innervated by the Vth nerve, the hyoid muscles by the VIIth nerve, and the branchial muscles by the IXth and Xth nerves. His epibranchial and hypobranchial muscles are "developed from the anterior myotomes of the body" and thus "are intrusive elements of the head"; they "retain a spinal innervation" and "do not receive any branches from the Vth, VIIth, IXth and Xth nerves". It should, however, be noted that recent developmental data has shown that head mesoderm is initially unsegmented and that, contrary to Edgeworth's hypothesis, it may not generate a migratory population of myoblasts as is the case in the anterior somites which give rise to the hypobranchial muscles. Instead, the mandibular, hyoid and branchial muscles appear to develop in situ under the influence of cephalic neural crest cells [30-33].

The main aim of the present paper is to provide a solid basis for future molecular, evolutionary and developmental work concerning zebrafish cranial musculature, by addressing four main questions: 1) How do the mandibular, hyoid and hypobranchial muscles of zebrafish develop until they reach their adult form? 2) To which muscles of other osteichthyans do these muscles correspond? 3) Is there a correspondence between the ontogeny of these muscles in the zebrafish and their evolutionary history within the Osteichthyes? 4) Regarding these cranial muscles, is it appropriate to consider the zebrafish as a "good representative" of teleosts, of actinopterygians and/or of bony fishes?

## Results

We examined the mandibular, hyoid and hypobranchial muscles of Edgeworth [12], i.e. the 'superficial cranial muscles' of Diogo and Vandewalle [37]. With exception to those few cases in which it is stated otherwise, our observations of these muscles in the early larvae analyzed mostly agree with those done in previous works [3,5,8-10]. The larval branchial and ocular muscles of the zebrafish have been described [3,5-10]. The configuration of the ocular muscles in adults is essentially similar to that found in larvae (data not shown).

### Mandibular musculature

**Figure 2:**
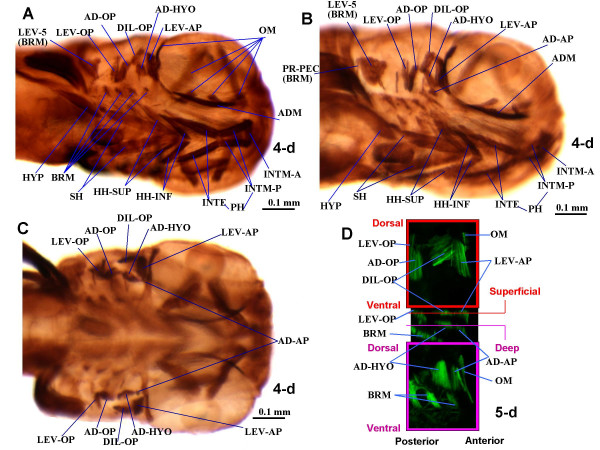
**Larval musculature of the zebrafish head**. Ventrolateral (**A, B, **showing different angles and certain distinct structures) and dorsal (**C**) views of immunohistochemical detection of myosin heavy chain in the cephalic muscles of 4-d zebrafish larvae (3.0 mm TL). Anterior to right. **D. **Confocal images showing green fluorescent protein (GFP) in the muscles adductor arcus palatini, adductor hyomandibulae, dilatator operculi and levator arcus palatini of a 5-d transgenic zebrafish larva expressing a GFP reporter driven from the muscle-specific alpha-actin promoter. Upper and lower panels: XY confocal optical sections through superficial and deep musculature, respectively. Central panel: XZ confocal reconstruction showing the plains of the confocal XY sections. AD-AP, adductor arcus palatini; AD-HYO, adductor hyomandibulae; AD-OP, adductor operculi; ADM, adductor mandibulae; BRM, branchial muscles; DIL-OP, dilatator operculi; HH-INF, hyoideus inferior; HH-SUP, hyoideus superior; HYP, hypaxialis; INTE, interhyoideus; INTM-A, INTM-P, intermandibularis anterior and posterior; LEV-AP, levator arcus palatini; LEV-OP, levator operculi; LEV-5, levator arcus branchialis 5; OM, ocular muscles; PR-H, protractor hyoideus; PR-PEC, protractor pectoralis; SH, sternohyoideus.

According to Schilling and Kimmel, five bilateral mandibular muscles innervated by the Vth nerve are formed in the first three days of zebrafish development: the intermandibularis anterior, the intermandibularis posterior, the adductor mandibulae, the levator arcus palatini and the dilatator operculi [3]. In their study, the adductor mandibulae began myosin protein expression at 53 hours post fertilization (53 hpf), the other four mandibular muscles appearing at 62 hpf [3]. These five muscles were found in the 4 day old (4-d) larvae examined in the present work (Fig. 2).

**Figure 3:**
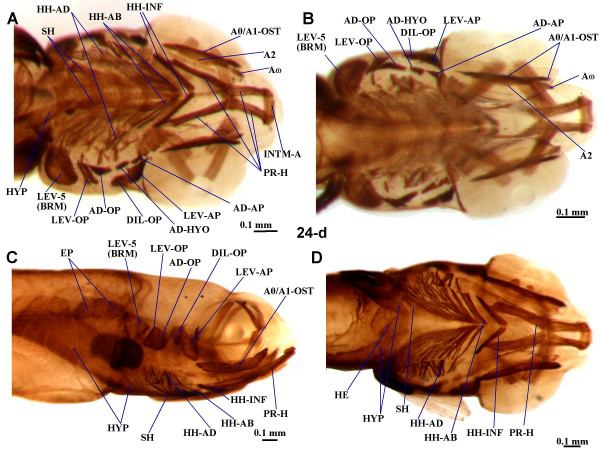
**Late larval musculature of zebrafish head**. Ventral (**A**) and dorsal (**B**) views of the cephalic muscles of 24-d zebrafish larvae (6.0 mm TL) and lateral (**C**) and ventral (**D**) views of the cephalic muscles and of the anterior portion of the body musculature of 24-d zebrafish larvae (6.9 mm TL). A0, A1-OST, A2, A0, AW, sections A0, A1-OST, A2, A0 and Aω of adductor mandibulae complex; AD-AP, adductor arcus palatini; AD-HYO, adductor hyomandibulae; AD-OP, adductor operculi; BRM, branchial muscle; DIL-OP, dilatator operculi; EP, epaxialis; HE, heart; HH-AB, hyoideus abductor; HH-AD, hyoidei adductores; HH-INF, hyoideus inferior; HYP, hypaxialis; INTM-A, intermandibularis anterior; LEV-AP, levator arcus palatini; LEV-OP, levator operculi; LEV-5, levator arcus branchialis 5; PR-H, protractor hyoideus; PR-H-D, PR-H-D, dorsal and ventral parts of protractor hyoideus; SH, sternohyoideus.

**Figure 4:**
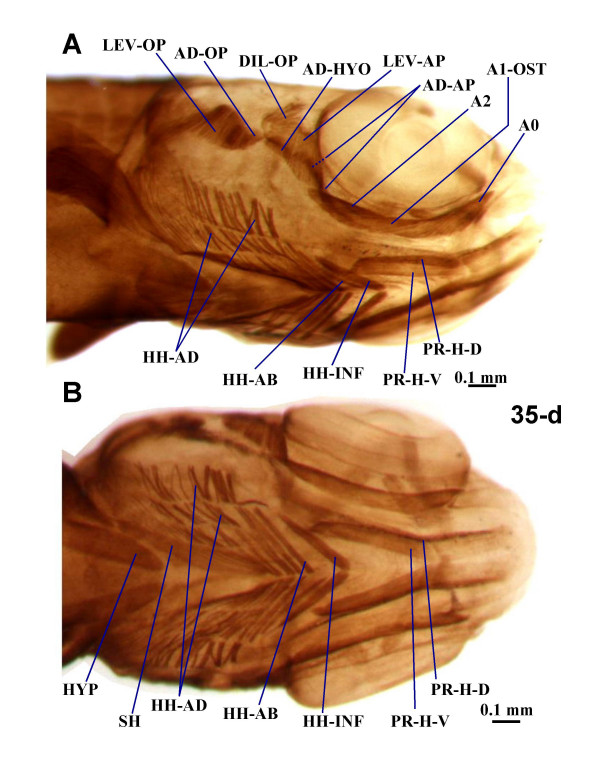
**Juvenile musculature of zebrafish head**. Ventrolateral (**A**) and ventral (**B**) views of the cephalic muscles of 35-d zebrafish larvae (7.4 mm TL). A0, A1-OST, A2, sections A0, A1-OST, and A2 of adductor mandibulae complex; AD-AP, adductor arcus palatini; AD-HYO, adductor hyomandibulae; AD-OP, adductor operculi; DIL-OP, dilatator operculi; HH-AB, hyoideus abductor; HH-AD, hyoidei adductores; HH-INF, hyoideus inferior; HYP, hypaxialis; LEV-AP, levator arcus palatini; LEV-OP, levator operculi; PR-H-D, PR-H-D, dorsal and ventral parts of protractor hyoideus; SH, sternohyoideus.

**Figure 5:**
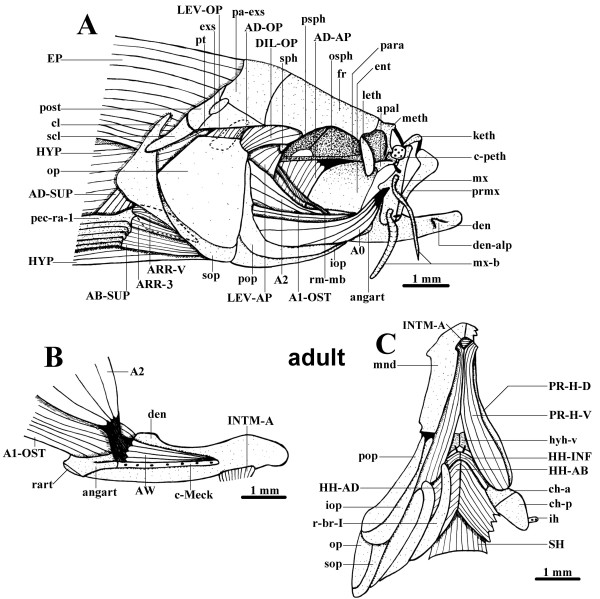
**Adult cranial musculature**. **A. **Lateral view of the cranial cephalic muscles and surrounding skeletal structures of an adult zebrafish (45.1 mm TL). **B. **Mesial view of the left mandible and adductor mandibulae of an adult zebrafish (45.1 mm TL), part of the anterior intermandibularis is also shown, the adductor mandibulae A0 was removed. **C. **Ventral view of the cephalic muscles and surrounding skeletal structures of an adult zebrafish (45.1 mm TL), on the right side a portion of the hyohyoidei adductores, as well as of the mandible, was cut, and the opercle, interopercle, subopercle and preopercle are not represented. A0, A1-OST, A2, AW, sections A0, A1-OST, A2 and Aω of the adductor mandibulae; AB-SUP, abductor superficialis; AD-AP, adductor arcus palatini; AD-OP, adductor operculi; AD-SUP, adductor superficialis; angart, angulo-articular; apal, autopalatine; ARR-3, arrector 3; ARR-V, arrector ventralis; c-Meck, Meckelian cartilage; c-peth, pre-ethmoid cartilage; ch-a, ch-p, anterior and posterior ceratohyals; cl, cleithrum; den, dentary bone; den-alp, anterolateral process of dentary bone; DIL-OP, dilatator operculi; ent, entopterygoid; EP, epaxialis; exs, extrascapular; fr, frontal; HH-AB, hyohyoideus abductor; HH-AD, hyohyoidei adductores; HH-INF, hyohyoideus inferior; hyh-v, ventral hypohyal; HYP, hypaxialis; ih, interhyal; INTM-A, intermandibularis anterior; iop, interopercle; keth, kinethmoid; leth, lateral-ethmoid; LEV-AP, levator arcus palatini; LEV-OP, levator operculi; meth, mesethmoid; mnd, mandible; mx, maxilla; mx-b, maxillary barbel; op, opercle; osph, orbitosphenoid; pa-exs, parieto-extrascapular; para, parasphenoid; pec-ra-1, pectoral ray 1; pop, preopercle; post, posttemporal; prmx, premaxilla; PR-H-D, PR-H-V, dorsal and ventral sections of protractor hyoidei; psph, pterosphenoid; pt, pterotic; r-br-I, branchiostegal ray I; rart, retroarticular; rm-mb, mesial branch of ramus mandibularis; scl, supracleithrum; SH, sternohyoideus; sop, subopercle; sph, sphenotic.

During development, the intermandibularis posterior becomes deeply associated with the hyoid muscle interhyoideus, forming the protractor hyoideus (Figs. 2A, B, 3, 4 and 5C). In contrast to earlier stages (Figs. 2A, B and 3A), in 35-d juveniles and adults two protractor hyoideus portions, dorsal and ventral, can be recognized (Figs. 4A, B and 5C). In adults, the ventral portion connects the anterior ceratohyal and ventral hypohyal bones to the ventromesial surface of the dentary bone of the mandible. The dorsal portion runs from the anterior ceratohyal to the ventromesial margin of the dentary bone (Table 1). Thus, the protractor hyoideus is a complex muscle innervated by both the Vth and VIIth nerves that results from a fusion of the posterior portion of the intermandibularis posterior to the anterior portion of the interhyoideus, followed by a longitudinal splitting to generate dorsal and ventral portions. This muscle is usually, but not always, associated with the elevation (protraction) of the hyoid bars, as well as with the depression of the mandible [38]. The overall configuration of the intermandibularis anterior remains rather unchanged during development. In adults this muscle connects the two dentary bones, thus joining the two mandibles (Figs. 2A, B, 3A and 5B, C; Table 1).

**Table 1:** Brief summary of the mandibular (man), hyoid (hyo) and hypobranchial (hyp) muscles found in the adult zebrafish, their attachments and main functions.

Name	Origin	Insertion	Function
**Intermandibularis anterior **(man)	dentary bone (mandible)	dentary bone of other side of body (mandible)	joins the two mandibles
**Protractor hyoideus **(man + hyo: intermandibularis posterior + interhyoideus)	ventral and dorsal portions: ventromesial surface of dentary bone (mandible)	ventral portion: anterior ceratohyal and ventral hypohyal; dorsal portion: anterior ceratohyal (hyoid arch)	mainly elevation of hyoid bars, as well as depression of mandible (mouth opening)
**Adductor mandibulae A2 **(man)	preopercle, hyomandibula and metapterygoid (suspensorium)	coronomeckelian bone (mandible)	the adductor mandibulae complex is mainly related with mouth closure, but the maxillary component A0 can also play a central role in the mouth protrusion mechanisms of the zebrafish (see text)
**Adductor mandibulae A1-OST **(man)	preopercle and quadrate (suspensorium)	angulo-articular and dentary bone (mandible)	
**Adductor mandibulae A0 **(man)	preopercle and quadrate (suspensorium)	maxilla (upper jaw)	
**Adductor mandibulae Aω **(man)	mesial surface of angulo-articular and dentary bone (mandible)	tendon of adductor mandibulae A2	
**Levator arcus palatini **(man)	sphenotic (neurocranium)	metapterygoid and hyomandibula (suspensorium)	suspensorial elevation/abduction
**Dilatator operculi **(man)	frontal and pterotic (neurocranium) and hyomandibula (suspensorium)	anterodorsal surface of opercle	opercular abduction (opening)
**Hyohyoideus inferior **(hyo)	anterior ceratohyals (hyoid arch)	mesial aponeurosis, meeting its contralateral counterpart	adduction of the hyoid arch (see text)
**Hyohyoideus abductor **(hyo)	first branchiostegal ray	mesial aponeurosis, meeting its contralateral counterpart	expansion of branchiostegal membrane
**Hyohyoidei adductores **(hyo)	opercle and subopercle	branchiostegal rays	constriction of branchiostegal membrane
**Adductor operculi **(hyo)	pterotic (neurocranium)	posterodorsal surface of opercle	opercular adduction (closure)
**Adductor arcus palatini **(hyo)	parasphenoid (neurocranium)	mesial sides of hyomandibula, metapterygoid and entopterygoid (suspensorium)	suspensorial adduction
**Adductor hyomandibulae X **(hyo)	parasphenoid (neurocranium)	mesial side of hyomandibula (suspensorium)	hyomandibular adduction
**Levator operculi **(hyo)	ventrolateral margin of pterotic (neurocranium)	dorsomesial edge of opercle	jaw depression (its force of contraction is transmitted through the opercular series and the interoperculo-mandibular ligament to the lower jaw: see text)
**Sternohyoideus **(hyp)	anterior region of cleithrum (pectoral girdle)	urohyal (associated with hyoid arch)	plays a major role in hyoid depression, and, through a series of mechanical linkages, in mouth opening and suspensorial abduction

As described in 5 and 6-d larvae [3,8-10], in the 4 and 9-d larvae observed the adductor mandibulae is constituted by a single mass of fibers (Fig. 2A, B). However, in the 14 and 24-d larvae examined, three different adductor mandibulae portions can be recognized (Fig. 3A, B and data not shown): they seemingly correspond to the adductor mandibulae A2, the Aω, and the A0/A1-OST of adults (of Diogo and Chardon [39]; Fig. 5A, B). In 35-d juveniles there is apparent separation between the adductor mandibulae A1-OST and the A0, the fibers of the former being more horizontally oriented and those of the latter being more anterodorsally oriented and inserting on the maxilla (Fig. 4A, compare with Fig. 5A). In adults, these two portions are well-differentiated (Fig. 5A). The overall configuration of the adult adductor mandibulae, divided into four portions, is thus rather different from the undivided adductor mandibulae found in early larvae. In adults, the adductor mandibulae A0 runs from the preopercle and quadrate to the maxilla (Fig. 5A). The adductor mandibulae A1-OST, mesial to the A0, runs from the preopercle and quadrate to the posterodorsal margin of the mandible, namely to the angulo-articular and the dentary bones (Fig. 5A, B). The adductor mandibulae A2 (Fig. 5A, B) is mesial to the A1-OST and connects the preopercle, hyomandibula and metapterygoid to the small coronomeckelian bone lodged on the mesial surface of the mandible. The adductor mandibulae Aω attaches anteriorly on the mesial surface of the angulo-articular and dentary bones and posteriorly on the tendon of the A2 (Fig. 5B). As its name indicates, the adductor mandibulae is mainly related with the adduction of the mandible (Table 1). However, it should be noted that since the adult A0 is attached on the maxilla and not on the mandible, it does not directly drive mandibular adduction. It is instead directly associated with adduction of the maxilla, and thus of the upper jaw, participating in the peculiar mechanisms of mouth protraction/retraction found in the zebrafish and other extant cypriniforms [11,20,14-16,39,40]. Thus, the larval adductor mandibulae undergoes splitting to generate different portions of diverse function in the adult.

Each of the two dorsal mandibular muscles of the zebrafish, the levator arcus palatini and dilatator operculi, remains undivided throughout the ontogenetic stages observed (Figs. 2, 3, 4 and 5A). In adults the levator arcus palatini (Fig. 5A) connects the sphenotic to the metapterygoid and hyomandibula and promotes the elevation/abduction of the suspensorium (a structural complex formed by the hyomandibula, quadrate and pterygoid bones) [38]. The dilatator operculi is lateral to the levator arcus palatini and connects the frontal, pterotic and hyomandibula to the anterodorsal surface of the opercle; it is mainly associated with opercular abduction (Fig. 5A; Table 1).

### Hyoid musculature

Five paired hyoid muscles are formed in the first four days of development: the interhyoideus, the hyohyoideus, the adductor hyomandibulae, the adductor operculi, and the levator operculi. The interhyoideus and hyohyoideus appear at 58 hpf, the adductor hyomandibulae and adductor operculi at 68 hpf, and the levator operculi at 85 hpf [3]. These five muscles, innervated by the VIIth nerve, are found in the larvae, juveniles and adults examined in the present work (Figs. 2, 3, 4 and 5A, C). As mentioned above, in the zebrafish, as in most other teleosts, the interhyoideus becomes associated with the intermandibularis posterior, forming the protractor hyoideus.

As in most other teleosts, in the adult zebrafish the hyohyoideus is innervated by the VIIth nerve, indicating its second arch origin [[3,13,38,40]; data not shown]. Hyohyoideus is divided into three paired structures: the hyohyoideus inferior runs from the anterior ceratohyals to a mesial aponeurosis in which it meets its contralateral counterpart; the hyohyoideus abductor runs from the first branchiostegal ray to a mesial aponeurosis that is attached by means of two thin tendons to the ventral hypohyals and in which it meets its contralateral counterpart; the hyohyoidei adductores connects the branchiostegal rays, the opercle and the subopercle of a single side of the fish (Fig. 5C; Table 1). As stated by Stiassny [38] "there is little commentary in the literature regarding the function of the hyohyoideus inferior but adduction of the hyoid bar is suggested by its position and presumed line of action". Regarding the hyohyoideus abductor and hyohyoidei adductores, which are often considered as parts of a hyohyoideus superior, they are usually associated with the expansion and constriction of the branchiostegal membranes, respectively (Fig. 5C; Table 1). A reference point that is often used to distinguish the hyohyoideus abductor and the hyohyoidei adductores is the position with respect to the most mesial branchiostegal ray: the hyohyoideus abductor is mesial to it and the hyohyoidei adductores lateral (Fig. 5C) [13]. At 9, 14, 24 and 35-d, the hyohyoideus is already separated into three portions that appear to correspond to the hyohyoideus inferior, hyohyoideus abductor and hyohyoidei adductores in adults (Figs. 3, 4, 5C and data not shown). In the 4-d larvae examined, the hyohyoideus is clearly divided into an anterior part, the hyohyoideus inferior, and a posterior part, named here hyohyoideus superior, but it is unclear if this latter is already differentiated into hyohyoideus abductor and hyohyoidei adductores (Figs. 2A, B and data not shown). In some zebrafish specimens, ossification of the branchiostegal rays (dermal bones directly derived from connective tissue) occurs by 4-d while in others it occurs later; it is thus likely that some 4-d larvae already have a hyohyoideus superior divided into hyohyoideus abductor and hyohyoidei adductores (separated by the most mesial branchiostegal ray) and others not [2].

The dorsal hyoid muscles adductor operculi and levator operculi are well separated throughout the zebrafish stages examined (Figs. 2, 3, 4 and 5A). The adductor operculi of adults lies mesial to the levator operculi and connects the pterotic to the posterodorsal surface of the opercle (Fig. 5A; Table 1). As its name indicates, it is mainly associated with opercular adduction (i.e. closure) [38]. The adult levator operculi runs from the ventrolateral margin of the pterotic to the dorsomesial edge of the opercle (see Fig. 5A; Table 1). The action of the levator operculi of teleosts is usually related to a peculiar mechanism mediating lower jaw depression via the so-called 'four-bar linkage system' in which the force of contraction of this muscle is transmitted through the opercular series (opercle, preopercle and/or interopercle) and the interoperculo-mandibular ligament to the lower jaw [38]. Our observations of zebrafish larvae and adults indicate that the action of the zebrafish levator operculi is similar to that found in other teleosts.

In addition to the five hyoid muscles described by Schilling and Kimmel [3], we observed in all specimens examined (from 4-d larvae to adults), an additional muscle, the adductor arcus palatini (Figs. 2B, C, 3A–B, 4A and 5A) [13]. In the other bony fishes in which this muscle is found, it is hyoid arch derived (see Tables 2, 3, 4, 5, 6, 7). In the adult zebrafish, the adductor arcus palatini runs from the neurocranium to the mesial sides of the hyomandibula, metapterygoid and entopterygoid (Fig. 5A; Table 1). It is anterior to, and broader than, the adductor hyomandibulae, which connects the neurocranium to the mesial margin of the hyomandibula. To confirm this observation, we examined 5-d alpha-actin GFP transgenic zebrafish larvae by confocal microscopy. The adductor arcus palatini and the adductor hyomandibulae effectively constitute separate muscles, which are often hidden behind the dilator operculi and levator arcus palatini in whole mount preparations (Fig. 2D). Although in the larval and juvenile specimens observed in the present work the adductor arcus palatini and adductor hyomandibulae lie close to each other, they also constitute distinct muscles (Figs. 2, 3A–B, and 4A). Our observations indicate that the action of the zebrafish adductor arcus palatini is somewhat similar to that found in other teleosts: it promotes the adduction of the suspensorium (a structural complex formed by the hyomandibula, quadrate and pterygoid bones), thus acting as the antagonist of the levator arcus palatini (see above) [38].

**Table 2:** Mandibular muscles of adults of representative actinopterygian taxa, including the zebrafish. The nomenclature of the muscles shown in bold follows that of the present work, "ad. mand." meaning adductor mandibulae. In order to facilitate comparisons, in some cases certain names often used by other authors to designate a certain muscle/bundle are given in front of that muscle/bundle. Data compiled from evidence provided by developmental biology, comparative anatomy, functional morphology, palaeontology, experimental embryology and molecular biology, innervation and phylogeny (for more details, see text).

Probable plesiomorphic osteichthyan condition	Cladistia: *Polypterus bichir *(Bichir)	Chondrostei: *Psephurus gladius *(Chinese swordfish)	Ginglymodi: *Lepisosteus osseus *(Longnose gar)	Halecomorphi: *Amia calva *(Bowfin)	Teleostei – basal: *Elops saurus *(Ladyfish)	Teleostei – clupeocephalan: *Danio rerio *(Zebrafish)
**Intermandibularis posterior**(*intermandibularis anterior and posterior plesiomorphically present in osteichthyans? See text)	**Intermandibularis**	**Intermandibularis**	**Intermandibularis**	**Intermandibularis posterior**	**Intermandibularis posterior **(*forming, together with interhyoideus, the protractor hyoideus)	**Intermandibularis posterior **(*see cell on the left)
**Intermandibularis anterior **(*see cell above)	-----	-----	-----	**Intermandibularis anterior**	**Intermandibularis anterior**	**Intermandibularis anterior**
-----	-----	-----	-----	-----	**Protractor hyoideus **(*including intermandibularis posterior and interhyoideus; it is thus derived from both the mandibular and hyoid muscle plates)	**Protractor hyoideus **(*see cell on the left)
**Ad. mand. A3'**	**Ad. mand. A3' **(ad. mand. of e.g. Lauder [69])	-----	**Ad. mand. A3' **(preorbitalis superficialis of e.g. Lauder [69])	**Ad. mand. A3'**	-----	-----
**Ad. mand. A3"**	**Ad. mand .A3" **(ad. mand. pterygoideus of e.g. Lauder [69])	-----	**Ad. mand. A3" **(preorbitalis profundus of e.g. Lauder [69])	**Ad. mand. A3"**	-----	-----
**Ad. mand. A2**	**Ad. mand. A2 **(ad. mand. posterolateral of e.g. Lauder [69])	**Ad. mand. A2 **(ad. mand. of e.g. Carroll and Wainwright [72])	**Ad. mand. A2 **(ad. mand. posterolateral of e.g. Lauder [69])	**Ad. mand. A2**	**Ad. mand. A2**	**Ad. mand. A2**
-----	-----	-----	**Palatomandibularis minor and major**	-----	-----	-----
-----	-----	-----	-----	**Levator maxillae superioris 3 and 4**	-----	-----
-----	-----	-----	-----	-----	-----	**Ad. mand. A1-OST**
-----	-----	-----	-----	-----	-----	**Ad. mand. A0**
**Ad. mand. Aω**	**Ad. mand. Aω**	-----	-----	**Ad. mand. Aω**	**Ad. mand. Aω**	**Ad. mand. Aω**
**Levator arcus palatini**	**Levator arcus palatini**	**Protractor hyomandibulae **(*seemingly originated from the portion of the hyoid muscle plate from which originate the adductor arcus palatini and dilatator operculi of other actinopterygians)	**Levator arcus palatini**	**Levator arcus palatini**	**Levator arcus palatini**	**Levator arcus palatini**
-----	**Dilatator operculi**	----- (*dilatator operculi absent as a separate element, but see cell above)	**Dilatator operculi**	**Dilatator operculi**	**Dilatator operculi**	**Dilatator operculi**

**Table 3:** Mandibular muscles of adults of representative sarcopterygian taxa. The nomenclature of the muscles shown in bold follows that of the present work, "ad. mand." meaning adductor mandibulae. In order to facilitate comparisons, in some cases certain names often used by other authors to designate a certain muscle/bundle are given in front of that muscle/bundle. Data compiled from evidence provided by developmental biology, comparative anatomy, experimental embryology and molecular biology, innervation and phylogeny (for more details, see text).

Probable plesiomorphic osteichthyan condition	Actinistia: *Latimeria chalumnae *(Coelacanth)	Dipnoi: *Lepidosiren paradoxa *(South American lungfish)	Amphibia: *Ambystoma ordinarium *(Michoacan stream salamander)	Reptilia: *Timon lepidus *(Ocellated lizard)	Mammalia: *Rattus norvegicus *(Norway rat)	Mammalia: *Homo sapiens *(Human)
**Intermandibularis posterior **(*see Table 2)	**Intermandibularis posterior**	**Intermandibularis**	**Intermandibularis posterior**	**Intermandibularis posterior**	**Mylohyoideus **(*mylohyoideus and digastricus anterior of rats derived from intermandibularis posterior, see e.g. Jarvik [53])	**Mylohyoideus**
**---**	**---**	**---**	**---**	**---**	**Digastricus anterior **(*see cell above)	**Digastricus anterior**
**Intermandibularis anterior **(*see Table 2)	**Intermandibularis anterior**	-----	**Intermandibularis anterior**	**Intermandibularis anterior**	**Intermandibularis anterior **(transversus mandibularis of Greene [83])	**---**
**Ad. mand. A3'**	**Ad. mand. A3' **(ad. mand. 'moyen' of e.g. Millot and Anthony [24])	**Ad. mand. A3' **(ad. mand. anterior of e.g. Bemis and Lauder [50])	**Ad. mand. A3' **(pseudotemporalis posterior of e.g. Iordansky [73])	**Ad. mand. A3' **(pseudotemporalis superficialis of e.g. Abdala and Moro [74])	**--- **(*but see below)	**--- **(*but see below)
**Ad. mand. A3"**	**Ad. mand. A3" **(ad. man. 'profond' of e.g. Millot and Anthony [24])	-----	**Ad. mand. A3" **(pseudotemporalis anterior of e.g. Iordansky [73])	**Ad. mand. A3" **(pseudotemporalis profundus of e.g. Abdala and Moro [74])	**--- **(*but see below)	**--- **(*but see below)
-----	-----	-----	-----	**Pterygomandibularis **(*seemingly derived from mesial portion of ad. mand.)	**--- **(*but see below)	**--- **(*but see below)
---	---	---	---	**---**	**Pterygoideus medialis **(*derived from mesial portion of ad. mand. of other tetrapods; seemingly corresponding to A3'/A3" and/or pterygomandibularis of e.g. lizards: see e.g. Saban [79])	**Pterygoideus medialis**
**Ad. mand. A2**	**Ad. mand. A2 **(ad. mand. 'superficiel' of e.g. Millot and Anthony [24])	**Ad. mand. A2 **(part of ad. mand. posterior of e.g. Bemis and Lauder [50])	**Ad. mand. A2 **(ad. mand. externus of e.g. Iordansky [73])	**Ad. mand. A2 **(ad. mand. externus of e.g. Abdala and Moro [74])	**Masseter **(*masseter, pterygoideus lateralis and temporalis of mammals seemingly derived from lateral, and eventually also medial, portions of ad. mand. of other tetrapods [12, 46, 79, 80])	**Masseter**
**---**	**---**	**---**	**---**	**---**	**Temporalis **(*see cell above)	**Temporalis**
**---**	**---**	**---**	**---**	**---**	**Pterygoideus lateralis **(*see cell above)	**Pterygoideus lateralis**
-----	-----	**Ad. mand. A2-PVM **(part of ad. mand. posterior of e.g. Bemis and Lauder [50])	**Ad. mand. A2-PVM **(ad. mand. posterior of e.g. Iordansky [73])	**Ad. mand. A2-PVM **(ad. mand. posterior of e.g. Abdala and Moro [74])	**Tensor veli palatini **(*tensor veli palatini and tensor tympani of mammals seemingly derived from ad. mand. A2-PVM of other tetrapods [12, 79])	**Tensor veli palatini**
---	---	**---**	**---**	**---**	**Tensor tympani **(*see cell above)	**Tensor tympani**
-----	-----	**Retractor anguli oris **(*seemingly derived from lateral portion of ad. mand.)	-----	-----	---	---
-----	-----	-----	-----	**Levator anguli oris **(*present, somewhat mixed with A2; seemingly derived from lateral portion of ad. mand, it may eventually be derived/modified from the retractor anguli oris, or at least from the portion of the mandibular muscle plate originating that muscle in other osteichthyan taxa)	**---**	**---**
**Ad. mand. Aω**	**Ad. mand. Aω **(intramandibular adductor of e.g. Lauder [75])	-----	-----	**Ad. mand. Aω **(*in *Timon *the ad. mand. has an anteroventral section that is lodged in the 'adductor fossa' and that is very similar to the Aω of other osteichthyan taxa: is this section homologous to the Aω of those taxa? [36])	**---**	**---**
**Levator arcus palatini**	**Levator arcus palatini**	-----	-----	**Levator pterygoidei **(*it may well be derived/modified from the levator arcus palatini, or at least from the portion of the mandibular muscle plate originating that muscle in other osteichthyan taxa)	**---**	**---**
-----	-----	-----	-----	**Protractor pterygoidei **(*same as cell above)	**---**	**---**

**Table 4:** Hyoid muscles of adults of representative actinopterygian taxa, including the zebrafish. The nomenclature of the muscles shown in bold follows that of the present work. In order to facilitate comparisons, in some cases certain names often used by other authors to designate a certain muscle/bundle are given in front of that muscle/bundle. Data compiled from evidence provided by developmental biology, comparative anatomy, experimental embryology and molecular biology, innervation and phylogeny (for more details, see text).

Probable plesiomorphic osteichthyan condition	Cladistia: *Polypterus bichir *(Bichir)	Chondrostei: *Psephurus gladius *(Chinese swordfish)	Ginglymodi: *Lepisosteus osseus *(Longnose gar)	Halecomorphi: *Amia calva *(Bowfin)	Teleostei – basal: *Elops saurus *(Ladyfish)	Teleostei – clupeocephalan: *Danio rerio *(Zebrafish)
**Interhyoideus**	**Interhyoideus**	**Interhyoideus**	**Interhyoideus**	**Interhyoideus**	**Interhyoideus **(*forming, together with intermandibularis posterior, the protractor hyoideus: see Table 2)	**Interhyoideus **(*see cell on the left)
-----	**Hyohyoideus**	**Hyohyoideus**	**Hyohyoideus**	**Hyohyoideus inferior**	**Hyohyoideus inferior**	**Hyohyoideus inferior**
-----	-----	-----	-----	**Hyohyoideus abductor **(*often considered as part of a hyohyoideus superior)	**Hyohyoideus abductor **(*see cell on the left)	**Hyohyoideus abductor **(*see cell on the left)
-----	-----	-----	-----	**Hyohyoidei adductores **(*often considered as part of a hyohyoideus superior)	**Hyohyoidei adductores **(*see cell on the left)	**Hyohyoidei adductores **(*see cell on the left)
**Adductor operculi**	**Adductor operculi**	**Adductor operculi **(opercularis of e.g. Carroll and Wainwright [72])	**Adductor operculi**	**Adductor operculi**	**Adductor operculi**	**Adductor operculi**
**Adductor arcus palatini**	**Adductor arcus palatini**	**Retractor hyomandibulae **(*seemingly originated from the portion of the hyoid muscle plate from which originates the adductor arcus palatini of other actinopterygians)	**Adductor arcus palatini**	**Adductor arcus palatini**	**Adductor arcus palatini**	**Adductor arcus palatini**
-----	-----	-----	-----	-----	-----	**'Adductor hyomandibulae X' **(*seemingly not homologous to the 'adductor hyomandibulae Y' of Table 5)
-----	-----	-----	-----	**Levator operculi **(*seemingly not homologous to the 'levator operculi' of Table 5)	**Levator operculi **(*see cell on the left)	**Levator operculi **(*see cell on the left)

**Table 5:** Hyoid muscles of adults of representative sarcopterygian taxa. The nomenclature of the muscles shown in bold follows that of the present work. In order to facilitate comparisons, in some cases certain names often used by other authors to designate a certain muscle/bundle are given in front of that muscle/bundle. Data compiled from evidence provided by developmental biology, comparative anatomy, experimental embryology and molecular biology, innervation and phylogeny (for more details, see text).

Probable plesiomorphic osteichthyan condition	Actinistia: *Latimeria chalumnae *(Coelacanth)	Dipnoi: *Lepidosiren paradoxa *(South American lungfish)	Amphibia: *Ambystoma ordinarium *(Michoacan stream salamander)	Reptilia: *Timon lepidus *(Ocellated lizard)	Mammalia: *Rattus norvegicus *(Norway rat)	Mammalia: *Homo sapiens *(Human)
**Interhyoideus**	**Interhyoideus **('géniohyoïdien' plus "hyohyoïdien' of e.g. Millot and Anthony [24])	**Interhyoideus**	**Interhyoideus **(interhyoideus anterior plus interhyoideus posterior of e.g. Bauer [57], and Ericsson and Olsson [32])	**Interhyoideus **(constrictor colli of e.g. Herrel et al [76])	**Part of facial muscles **(*facial muscles of mammals derive mostly from interhyoideus, but possibly also from cervicomandibularis, see e.g. Lightoller [81] and Saban [79)	**Part of facial muscles**
**Adductor arcus palatini**	**Adductor arcus palatini**	----- (*does the portion of the hyoid muscle plate that gives rise to the levator hyoideus/depressor mandibulae eventually correspond to that giving rise to the adductor arcus palatini of other osteichthyans?)	----- (*see cell on the left)	----- (*see on the left)	----- (*see on the left)	----- (*see on the left)
-----	-----	**Levator hyoideus**	**Depressor mandibulae posterior **(*the fibers corresponding to those of the levator hyoideus of dipnoans become also attached on the mandible, forming the depressor mandibulae posterior; the depressor mandibulae anterior thus seemingly corresponds to the depressor mandibulae of dipnoans)	**Depressor mandibulae (part) **(*the fibers corresponding to those of the levator hyoideus of dipnoans become also attached on the mandible, forming part of the depressor mandibulae)	--- (*but see below)	--- (*but see below)
-----	-----	**Depressor mandibulae**	**Depressor mandibulae anterior **(*see cell above)	**Depressor mandibulae (part) **(*see cell above)	--- (*but see below)	--- (*but see below)
-----	-----	-----	**Branchiohyoideus **(branchiohyoideus externus of e.g. Edgeworth [12] and Ericsson and Olsson [32], which is seemingly derived from levator hyoideus/depressor mandibulae)	----- (*as noted by e.g. Edgeworth [12] the 'branchiohyoideus' of lizards seemingly corresponds to the branchial muscle subarcualis rectus 1 of amphibians, and not to the hyoid muscle branchiohyoideus of the present work)	---	---
-----	-----	-----	-----	**Cervicomandibularis **(*seemingly derived from levator hyoideus/depressor mandibulae [12])	**--- **(*but see above)	**--- **(*but see above)
---	---	---	---	**---**	**Stapedius **(*stapedius of mammals derived from levator hyoideus/depressor mandibulae [12, 46, 82])	**Stapedius**
---	---	---	---	**---**	**Digastricus posterior **(*digastricus posterior and stylohyoideus of mammals seemingly derived from levator hyoideus/depressor mandibulae, and not from interhyoideus; see e.g. Huber [82])	**Digastricus posterior**
---	---	---	---	**---**	**Stylohyoideus **(*see cell above)	**Stylohyoideus**
-----	**'Adductor hyomandibulae Y' **(*seemingly not homologous to the 'adductor hyomandibulae X' of Table 4)	-----	-----	-----	---	---
**Adductor operculi**	**Adductor operculi**	----- (*absent as a separate element in adults, but see text)	-----	-----	---	---
-----	***Latimeria*'s 'levator operculi' **(*seemingly not homologous to the levator operculi of Table 4)	-----	-----	-----	---	---

**Table 6:** Hypobranchial muscles of adults of representative actinopterygian taxa, including the zebrafish. The nomenclature of the muscles shown in bold follows that of the present work. Data compiled from evidence provided by developmental biology, comparative anatomy, experimental embryology and molecular biology, innervation and phylogeny (for more details, see text).

Probable plesiomorphic osteichthyan condition	Cladistia: *Polypterus bichir *(Bichir)	Chondrostei: *Psephurus gladius *(Chinese swordfish)	Ginglymodi: *Lepisosteus osseus *(Longnose gar)	Halecomorphi: *Amia calva *(Bowfin)	Teleostei – basal: *Elops saurus *(Ladyfish)	Teleostei – clupeocephalan: *Danio rerio *(Zebrafish)
**Coracomandibularis**	**Branchiomandibularis **(*modified from coracomandibularis)	**Branchiomandibularis **(*see cell on the left)	-----	**Branchiomandibularis **(*see cells on the left)	-----	-----
**Sternohyoideus**	**Sternohyoideus**	**Sternohyoideus**	**Sternohyoideus**	**Sternohyoideus**	**Sternohyoideus**	**Sternohyoideus**

**Table 7:** Hypobranchial muscles of adults of representative sarcopterygian taxa. The nomenclature of the muscles shown in bold follows that of the present work. In order to facilitate comparisons, in some cases certain names often used by other authors to designate a certain muscle/bundle are given in front of that muscle/bundle. Data compiled from evidence provided by developmental biology, comparative anatomy, experimental embryology and molecular biology, innervation and phylogeny (for more details, see text).

Probable plesiomorphic osteichthyan condition	Actinistia: *Latimeria chalumnae *(Coelacanth)	Dipnoi: *Lepidosiren paradoxa *(South American lungfish)	Amphibia: *Ambystoma ordinarium *(Michoacan stream salamander)	Reptilia: *Timon lepidus *(Ocellated lizard)	Mammalia: *Rattus norvegicus *(Norway rat)	Mammalia: *Homo sapiens *(Human)
**Coracomandibularis**	**Coracomandibularis**	**Coracomandibularis **(geniothoracicus of e.g. Miyake et al. [34])	**Geniohyoideus **(*geniohyoideus does not correspond directly to coracomandibularis of bony fishes, because this latter also gave rise to tetrapod muscles as e.g. genioglossus and hyoglossus; so in this case we can accept to use the name geniohyoideus, due to its consensual use within anatomists working with tetrapods)	**Geniohyoideus **(geniohyoideus and/or at least part of mandibulohyoideus of e.g. Edgeworth [12] and Herrel et al. [76])	**Geniohyoideus**	**Geniohyoideus**
-----	-----	-----	**Genioglossus **(*according to e.g. Edgeworth [12] the genioglossus of salamanders such as *Ambystoma *is derived from the coracomandibularis)	**Genioglossus **(*according to e.g. Edgeworth [12] the genioglossus of lizards such as *Timon *is derived from the coracomandibularis)	**Genioglossus**	**Genioglossus**
-----	-----	-----	**Hyoglossus **(*the statements of Edgeworth [12] concerning the origin of this muscle in salamanders such as *Ambystoma *are somewhat confuse: in his page 196 he states that it originates from the sternohyoideus but in his page 211 he seems to indicate that, as in other amphibians as well as in amniotes, it derives from the coracomandibularis)	**Hyoglossus **(*according to e.g. Edgeworth [12] the hyoglossus of lizards such as *Timon *is derived from the coracomandibularis)	**Hyoglossus**	**Hyoglossus**
---	---	---	**---**	**---**	**Styloglossus **(*derived from hyoglossus, see e.g. Edgeworth [12] and Saban [79])	**Styloglossus**
---	---	---	**---**	**---**	**---**	**Palatoglossus **(*seemingly derived from styloglossus, see e.g. Edgeworth [12])
**Sternohyoideus**	**Sternohyoideus**	**Sternohyoideus **(rectus cervicis of e.g. Bemis and Lauder [50])	**Sternohyoideus **(rectus cervicis of e.g. Lauder and Shaffer [77])	**Sternohyoideus **(rectus cervicis of e.g. Kardong [46])	**Sternohyoideus**	**Sternohyoideus**
**---**	**---**	**---**	**---**	**---**	**Sternothyroideus **(*sternothyroideus and thyrohyoideus seemingly derived from sternohyoideus, see e.g. Edgeworth [12], Saban [79], and Kardong [46])	**Sternothyroideus**
**---**	**---**	**---**	**---**	**---**	**Thyrohyoideus **(*see cell above)	**Thyrohyoideus**
-----	-----	-----	**Omohyoideus **(*seemingly derived from the sternohyoideus)	**Omohyoideus**	**Omohyoideus**	**Omohyoideus**

### Hypobranchial musculature

There is a single hypobranchial muscle in the zebrafish: the sternohyoideus ([12]; Figs. 2A, B, 3A and 5C). In the study of Schilling and Kimmel [3] this muscle, innervated by the anterior branches of the occipito-spinal nerves, appeared at 53 hpf. In early stages the sternohyoideus is markedly divided longitudinally, its right and left parts only meeting anteriorly, near the region of the hyohyoideus inferior (Figs. 2A, B). As described in zebrafish and several other teleosts, each of these parts consists of three myomeres separated by two myocommata [3,12,13]. In older stages of development the right and left parts become closer to each other; in adults they are connected mesially throughout their lengths, forming a large cone-shaped structure that originates from the anterior region of the cleithrum and passes dorsally to the hyohyoideus inferior and hyohyoideus abductor in order to attach on the urohyal (Fig. 5C; Table 1). The sternohyoideus plays a major role in hyoid depression, and, through a series of mechanical linkages, in mouth opening and suspensorial abduction [8,38]. As in numerous other teleosts [12,13], the posterior portion of the sternohyoideus lies near the anterior attachment of the hypaxialis (ventral body musculature) on the pectoral girdle (Figs. 2A, B, 3A, and 4B), the fibers of the former being sometimes associated posteriorly with fibers of the latter (e.g. Fig. 2B). Therefore, the contraction of the hypaxialis during a feeding strike may not only prevent the origin of the sternohyoideus from moving anteriorly, but also facilitate a greater ventral displacement of the hyoid (= hyoid depression) by pulling the posterior portion of the sternohyoideus backwards [8]. In zebrafish larvae the anterior portion of the epaxial and hypaxial body muscles extend anteriorly to attachments on the back of the skull and pectoral girdle, thus lying near to, and eventually associating with, the head muscles (e.g. Fig. 3C, D). This configuration is also seen in the adult zebrafish (Fig. 5A).

## Discussion

### Homologies of the zebrafish mandibular, hyoid and hypobranchial muscles

As raised in the Background section, a major question addressed is the present paper is: to which muscles of other osteichthyans do the mandibular, hyoid and hypobranchial muscles of the zebrafish correspond? Here we add the zebrafish to the up-dated compilation of the extant data and discussion of the development, evolution and homologies of cranial muscles within various major groups of Osteichthyes [36]. Our discussion provides a starting point for investigating the identity and homologies between zebrafish mandibular, hyoid and hypobranchial muscles and the muscles found in other osteichthyans (Tables 2, 3, 4, 5, 6).

#### Mandibular muscles (Tables 2, 3)

According to Edgeworth [12], in numerous gnathostomes the embryonic mandibular muscle plate gives rise dorsally to the premyogenic condensation constrictor dorsalis and medially to the premyogenic condensation adductor mandibulae. To this can be added, ventrally, the intermandibularis (Tables 2, 3). Molecular developmental studies have supported the existence of the constrictor dorsalis in the cranial region of teleosts [7,35]. Expression of Engrailed genes marks muscle cells associated with the dorsal region of the first arch [7,35]. The constrictor dorsalis was plesiomorphically found in osteichthyans and then independently lost in dipnoans and amphibians (Tables 2, 3; see fig. 1). The constrictor dorsalis that gave rise to the levator arcus palatini and dilatator operculi in zebrafish and other actinopterygians is therefore homologous with the constrictor dorsalis that gives rise to e.g. the levator arcus palatini in extant sarcopterygian fishes such as *Latimeria *and to the protractor pterygoidei and levator pterygoidei in certain amniotes (Tables 2, 3).

Regarding the ventral portion of the mandibular muscle plate, in all major osteichthyan groups listed in Tables 2 and 3 it gives rise to the intermandibularis. In adult extant members of Actinistia, Chondrostei, Ginglymodi and Dipnoi the intermandibularis is mainly undivided. Adults of *Amia*, *Latimeria*, and numerous amphibian, amniote and teleostean genera, including *Danio*, exhibit an intermandibularis anterior and an intermandibularis posterior. It is, therefore, difficult to discern if the intermandibularis was divided or not in plesiomorphic adult osteichthyans (Tables 2, 3).

As in the zebrafish, in most teleosts the intermandibularis posterior and interhyoideus form the protractor hyoideus, which is thus derived from the mandibular and hyoid muscle plates (Figs. 2, 3, 4, and 5C). Although a protractor hyoideus is not found in a few teleosts such as *Albula *and *Mormyrus *[13,41,42], this muscle was seemingly present in the ancestors of extant teleosts (Table 2). Based on the altered morphology of the protractor hyoideus in morpholino-mediated Hox PG2 (*hoxa2b *and *hoxa2a*) knock-down larvae, Hunter and Prince [10] suggested that in the zebrafish "the basihyal (cartilage) may be important for the proper ontogenetic organization" of the intermandibularis posterior and the interhyoideus, and, thus, for the association of their fibers and the formation of the protractor hyoideus. Further studies are needed to check if this is so and if it is a general feature within the Teleostei.

The adductor mandibulae is found in members of all major osteichthyan groups (Tables 2, 3). The number of divisions of this muscle is highly variable within these groups (Tables 2, 3). As often occurs with other muscles, different names are used in the literature to designate the adductor mandibulae divisions in different osteichthyan taxa, and sometimes within the same taxon. This is a major reason for the historical confusions concerning the homologies and evolution of these divisions within osteichthyans. The names employed in Tables 2, 3, 4, 5, 6, 7 are those often used by researchers working with phylogenetically more plesiomorphic groups. Our observations regarding the development, function and adult configuration of the zebrafish adductor mandibulae A2 and Aω indicate that these sections correspond to the A2 and Aω found in most major osteichthyan groups. Regarding the zebrafish adductor mandibulae A1-OST and A0, these divisions correspond to adductor mandibulae sections that are exclusively found in ostariophysan teleosts and in cypriniforms, respectively [19,39]. Hernandez et al. [9] stated that the adult zebrafish has an adductor mandibulae Aω and three further sub-divisions, as we also observe. The adductor mandibulae A1, A2 and A3 of Hernandez et al. probably correspond, respectively, to the A0, A1-OST and A2 of the present study (see Table 2; Figs. 5A, B).

#### Hyoid muscles (Tables 4, 5)

Edgeworth [12] suggested that a constrictor hyoideus condensation usually gives rise to dorso-medial and ventral derivatives throughout the major groups of gnathostomes. Two dorso-medial hyoid muscles were seemingly found in plesiomorphic osteichthyans: the adductor arcus palatini and the adductor operculi (Tables 4, 5). These muscles are found in most teleosts, including the zebrafish (Figs. 2B, C, 3, 4, and 5A). A few teleosts lack an adductor operculi (e.g. saccopharyngiforms) [43-45]. Apart from the adductor arcus palatini and the adductor operculi, other dorso-medial hyoid muscles are found in certain living osteichthyans (Tables 4, 5). For example, the zebrafish, as most extant teleosts and the halecomorph *Amia*, has a muscle levator operculi (Figs. 2, 3, 4, and 5A). Millot and Anthony [24] stated that *Latimeria *has a 'levator operculi'. However, whether this muscle is homologous to the levator operculi of zebrafish is doubtful for two main reasons. First, the muscles have distinct function: contrary to the zebrafish and other teleosts and to *Amia*, *Latimeria *does not have an interoperculo-mandibular ligament and, therefore, does not have an opercular mechanism mediating mandible depression [36]. Second, and more importantly, it is cladistically more parsimonious to consider that these muscles were independently acquired in actinistians and halecostomes (2 steps) than to have one acquisition (in the node leading to osteichthyans) and various independent losses (at least in non-actinistian sarcopterygians, in cladistians, in chondrosteans and in ginglymodians) (see Fig. 1). On balance, our view is that the 'levator operculi' of *Latimeria *is unlikely to be homologous with the levator operculi of the zebrafish and other teleosts and of *Amia *(see Tables 4, 5). Similar reasoning applies to the 'adductor hyomandibulae' of *Latimeria *and to the adductor hyomandibulae found in the zebrafish and certain other teleosts (see below). However, further studies, and particularly more detailed palaeontological data, are needed to clarify the exact taxonomic distribution of these muscles within osteichthyans. The dipnoan 'levator operculi' illustrated by Kardong [46], which may correspond to the adductor operculi of other bony fishes but forms, in extant adult dipnoans, a continuous sheet of fibers together with other cranial muscles [12,21,23,47-51], seemingly corresponds to the constrictor operculi of Bemis and Lauder [50]. Therefore, the levator operculi found in the zebrafish, most other teleosts and *Amia *is seemingly not homologous with any of the individual cranial muscles of other extant osteichthyans: it probably derived evolutionarily from the adductor operculi at the node leading to the Halecostomi (Fig. 1, Table 4) [36].

Apart from the adductor arcus palatini, some osteichthyans have other muscles connecting the neurocranium to the palatoquadrate/suspensorium and promoting the adduction of these latter structures. This is the case in zebrafish, which exhibit an adductor arcus palatini and an adductor hyomandibulae according to Winterbottom's nomenclature [13]. There is much confusion in the literature concerning these muscles. As explained by Winterbottom [13], in most teleosts there is a single muscle connecting the neurocranium to the mesial surface of the suspensorium and thus acting to adduct this latter structure. Winterbottom opted to designate this muscle 'adductor arcus palatini' and not 'adductor hyomandibulae' because the latter name becomes inappropriate in the numerous taxa in which this muscle is expanded anteriorly along the floor of the orbit and attaches on elements of the suspensorium other than the hyomandibula, for example the metapterygoid and/or entopterygoid (as is the case in the adult zebrafish). He therefore used the name 'adductor hyomandibulae' to designate a muscle that is only found in a few osteichthyans (one of them being the zebrafish) and that is usually situated posteriorly to his adductor arcus palatini, connecting the neurocranium to the mesial surface of the hyomandibula. This nomenclature is followed in the present study (Tables 4, 5). At least some of the muscles 'adductor hyomandibulae' of osteichthyans are non-homologous, as they may originate "1) either from the posterior region of the adductor arcus palatini or 2) from the anterior fibers of the adductor operculi" [13]. This is for instance the case of the 'adductor hyomandibulae' found in *Latimeria *[24] and in various teleosts (Tables 4, 5; see above). In order to distinguish the 'adductor hyomandibulae' of the zebrafish from the 'adductor hyomandibulae' of *Latimeria*, these muscles are designated in Tables 4, 5 as 'adductor hyomandibulae X' and 'adductor hyomandibulae Y', respectively.

Examples of dorso-medial hyoid muscles that are not found in the zebrafish, but which are present in other osteichthyans, are the levator hyoideus and the depressor mandibulae, which seemingly gave rise to the stylohyoideus, digastricus posterior, stapedius, and possibly part of the facial muscles of mammals (Table 5). The levator hyoideus is usually related with the elevation of the posterodorsal portion of the ceratohyal, whereas the depressor mandibulae is usually related with the opening of the mouth [49,50]. The levator hyoideus is found in at least some developmental stages of extant dipnoans and of numerous extant tetrapods [12]. The depressor mandibulae of extant dipnoans such as *Lepidosiren *and *Protopterus *seems to be homologous with part of the depressor mandibulae of tetrapods (see Table 7). Interestingly, works such as Köntges and Lumsden [30] have shown that in tetrapod taxa such as birds the posterior region of the mandible to which the depressor mandibulae attaches is constituted by neural crest derivatives of the hyoid arch, and not of the mandibular arch. This is one of the several examples given by these authors to illustrate the highly constrained pattern of cranial skeletomuscular connectivity found in these tetrapods: each rhombomeric neural crest population remains coherent throughout ontogeny, forming both the connective tissues of specific muscles and their respective attachment sites onto the neuro- and viscerocranium. It would be interesting, therefore, to investigate if the depressor mandibulae of dipnoans such as *Protopterus *and *Lepidosiren *also attaches in a region of the mandible constituted by neural crest derivatives of the hyoid arch. If future investigation shows that the mandible of extant non-dipnoan bony fishes is exclusively formed by mandibular neural crest derivatives, this would indicate that the presence of a depressor mandibulae in tetrapods and dipnoans might be related with an evolutionary change in which hyoid neural crest derivatives have become incorporated in the formation of the lower jaw.

The plesiomorphic condition for osteichthyans is seemingly that in which the ventral portion of the hyoid muscle plate gives rise to a single division, designated here as interhyoideus (Tables 4, 5). In most extant actinopterygians part of the interhyoideus separates into a distinct muscle during development, the hyohyoideus (Table 4). In adult zebrafish, as in most other teleosts, the hyohyoideus is divided into hyohyoideus inferior, hyohyoideus abductor and hyohyoidei adductores (Fig. 5C; Tables 4, 5). Beyond teleosts, the presence of these three divisions is only found in the extant halecomorph *Amia *(Tables 4, 5). An independent hyohyoideus in seemingly missing in extant Sarcopterygii (Table 5). Although there are some sarcopterygians in which the portion of the hyoid muscle plate that gives rise to the interhyoideus and hyohyoideus in actinopterygians eventually becomes somewhat divided into bundles that resemble these two muscles, these bundles remain deeply mixed throughout all developmental stages. This is the case of the interhyoideus anterior and interhyoideus posterior of various salamanders (Table 5) [52-58]. It is seemingly also the case of the "géniohyoïdien" and "hyohyoïdien" described by Millot and Anthony [24] in *Latimeria *(Table 5). Further studies are needed to determine whether splitting of interhyoideus evolved repeatedly in osteichthyans.

#### Hypobranchial muscles (Tables 6, 7)

The plesiomorphic condition for osteichthyans is seemingly that found in adult members of Actinistia and Dipnoi, which exhibit two hypobranchial muscles: a coracomandibularis and a sternohyoideus (Tables 6, 7). In extant cladistians, chondrosteans and halecomorphs the coracomandibularis is modified into a peculiar muscle branchiomandibularis that connects the branchial arches to the mandible. A coracomandibularis/branchiomandibularis is missing in living ginglymodians and teleosts, including the zebrafish (Table 6). Therefore, contrary to what is sometimes stated in the literature, the geniohyoideus of tetrapods does not correspond to the zebrafish protractor hyoidei, nor to any of its constituents (i.e. the intermandibularis posterior and the interhyoideus) (Tables 6, 7). The absence of a coracomandibularis/branchiomandibularis in living ginglymodians and in teleosts is seemingly due to a secondary loss [59-61]. In extant tetrapods, there are various hypobranchial muscles that are not found in other extant osteichthyans, for example the omohyoideus, sternothyroideus, thyrohyoideus and the specialized glossal muscles related with the movements of the tongue (Table 7) [12,46,53,54,62-68].

### Ontogeny and Phylogeny

Another major question addressed by this paper is: does the development of the mandibular, hyoid and hypobranchial muscles in the zebrafish correspond to the evolution of these muscles within the Osteichthyes? Our analysis shows that only in certain cases is this true. For example, based on our previous cladistic analysis [36], within osteichthyan evolutionary history the mandibular muscles intermandibularis anterior, intermandibularis posterior, adductor mandibulae and levator arcus palatini were seemingly present in basal osteichthyans; the dilatator operculi was apparently only acquired later in evolution, being exclusively found in actinopterygians (Table 2). However, according to Schilling and Kimmel, sarcomeric myosin expression (SME; i.e. contractile function) of the dilatator operculi, levator arcus palatini, intermandibularis anterior and intermandibularis posterior begins ontogenetically at about the same time in the zebrafish, i.e. at 62 hpf (the adductor mandibulae appears at 53 hpf) [3]. In contrast, the development of the zebrafish adductor mandibulae divisions does seem to follow the order in which these divisions were acquired in evolution. The adductor mandibulae A2 and Aω were acquired first in evolution, being plesiomorphically found in osteichthyans; the adductor mandibulae A1-OST and A0 were acquired later, namely in the nodes leading to ostariophysans and to cypriniforms, respectively (Tables 2, 3) [36]. During zebrafish development, the adductor mandibulae A2 and Aω also form earlier, being already separated in the 9-d larvae examined. The adductor mandibulae A1-OST and A0 were only distinguished in 35-d juveniles and adults.

The order in which the hyoid muscles were acquired in evolution is: first, the interhyoideus, adductor operculi and adductor arcus palatini (plesiomorphically found in osteichthyans); then, the hyohyoideus (only found in extant actinopterygians); then, the levator operculi (only found in extant halecomorphs and teleosts); and, lastly, the adductor hyomandibulae X (found in some teleosts, seemingly not homologous with the adductor hyomandibulae Y of *Latimeria*) (Table 4) [36]. According to Schilling and Kimmel, in the zebrafish SME of the interhyoideus and hyohyoideus begins at 58 hpf, of the adductor operculi and adductor hyomandibulae at 68 hpf, and of the levator operculi at 85 hpf [3]. Thus, as in phylogeny, in the zebrafish SME of the levator operculi begins later than SME of the interhyoideus, the hyohyoideus and the adductor operculi. However, unlike phylogeny, SME of the zebrafish hyohyoideus begins earlier than the adductor operculi.

The single hypobranchial muscle in the zebrafish, the sternohyoideus, begins SME at 53 hpf and consists of left and right parts [3]. We show that these parts gradually fuse during later development. Interestingly, in adult basal actinopterygians, the sternohyoideus is longitudinally divided into left and right parts that remain physically separate [69]. This plesiomorphic configuration was, however, modified in the node leading to the Teleostei: in adult teleosts, including the zebrafish, the sternohyoideus is a cone-shaped structure in which the left and right parts are hardly distinguished from each other. Thus, during zebrafish development the overall configuration of the sternohyoideus becomes modified in a manner that resembles the changes that occurred in actinopterygian evolution.

The examples above show that although in certain cases there is a correspondence between the ontogeny of the mandibular, hyoid and hypobranchial muscles in the zebrafish and the evolution of these muscles within Osteichthyes, this is not always the case. This also applies to other zebrafish head muscles, as well as certain cartilages and bones. For example, as shown in Figure 2A, B, in 4-d zebrafish larvae the levator arcus branchialis 5 is already much broader than the other branchial muscles, prior to the splitting of the adductor mandibulae into different sections. However, in evolution the hypertrophy of the levator arcus branchialis 5 occurred only in the node leading to cypriniforms, much later than the division of the adductor mandibulae in different sections (Fig. 1; Table 2) [36]. The modification of the muscle levator arcus branchialis 5, as well as of the skeletal structure that is moved by this muscle, the ceratobranchial 5, is related with the specialized feeding mechanisms of cypriniforms [2,3,11-18]). Ceratobranchial 5 bears teeth and ossifies earlier than other ceratobranchials in cypriniforms, a case of 'acceleration' of development [3]. Such coordinated ontogenetic timing changes may ensure proper size relationships between skeletal and myological structures.

### Conclusion: zebrafish as a case study

The zebrafish is the most studied model organism among osteichthyan fishes, and is often taken as a 'good representative' of teleosts, of actinopterygians, and even of bony fishes in developmental and molecular studies. But, regarding its mandibular, hyoid and hypobranchial muscles, to what extent is it appropriate to consider the zebrafish as an appropriate 'representative' of these groups?

As can be seen in Tables 2, 3, 4, 5, 6, 7, all of the 13 mandibular, hyoid and hypobranchial muscles found in the adult zebrafish (intermandibularis anterior, protractor hyoideus, adductor mandibulae, levator arcus palatini, dilatator operculi, hyohyoideus inferior, hyohyoideus abductor, hyohyoidei adductores, adductor arcus palatini, adductor hyomandibulae, adductor operculi, levator operculi, and sternohyoideus) are found in at least some other living teleosts, and all except the protractor hyoideus are found in at least some non-teleost extant actinopterygians. Therefore, although the zebrafish occupies a rather derived phylogenetic position within the Actinopterygii and even within the Teleostei (Fig. 1), with respect to these muscles, it seems justified to consider the zebrafish as a potential representative of these two groups. Moreover, of these 13 muscles, about half are found in at least some extant sarcopterygian fishes (six muscles; namely the intermandibularis anterior, adductor mandibulae, levator arcus palatini, adductor arcus palatini, adductor operculi, and sternohyoideus). About a quarter can be confidently identified in at least some extant adult tetrapods (three muscles; the intermandibularis anterior, adductor mandibulae, and sternohyoideus). Therefore, among the cranial muscles discussed in this paper, these three latter muscles are particularly appropriate for direct comparisons between the results obtained in molecular and developmental studies of the zebrafish and the data obtained from model tetrapod organisms from clades such as Amphibia and/or Amniota. The information provided here forms a solid basis for future analyses on zebrafish cranial muscles and for a proper comparison between these muscles and those found in other osteichthyans.

## Methods

King's wild type or *Tg(acta1:GFP) *[70] zebrafish were reared according to Westerfield [71]. 4-d (96 hpf, 10 larvae, mean total length 3.2 mm), 9-d (216 hpf, 10 larvae, mean total length 4.0 mm), 14-d (336 hpf, 10 larvae, mean total length 4.5 mm), 24-d (576 hpf, 10 larvae mean total length 6.5 mm) and 35-d (986 hpf, 10 juveniles, mean total length 7.4 mm) were killed and fixed in 4% paraformaldehyde. Larval or juvenile fish were bleached in 1%H_2_O_2 _5% formamide solution to remove pigment, processed for immunohistochemistry with anti-myosin heavy chain antibody A4.1025 as previously described [3], and viewed and photographed on a Zeiss Axiophot. Adult specimens (10, from the collection of the Museo Nacional de Ciencias Naturales de Madrid, about 1-year-old, mean length 45.2 mm) were alcohol-preserved. Dissections and morphological drawings of adult specimens were made using a Wild M5 dissecting microscope equipped with a camera lucida. The nomenclature used to designate the skeletal and muscular structures follows that of Diogo [36]. The phylogenetic framework for the discussions provided in the present paper is based on the results of a recent cladistic analysis of osteichthyan higher-level phylogeny including 356 phylogenetic osteological and myological characters and 80 extant and fossil terminal taxa (Fig. 1) [36].

## Authors' contributions

RD and SMH designed the study, carried out the experiments, analyzed the data and drafted the manuscript. The configuration of adult zebrafish cranial muscles was analysed by RD. YH analysed alpha-actin GFP transgenic zebrafish larvae and obtained the confocal images. All the work was done in the MRC Centre for Developmental Neurobiology and the Randall Division for Cell and Molecular Biophysics. All authors read and approved the final manuscript.
